# Clinical evaluation of flat peripheral curve design with aspherical-curve and multi-curve hard contact lenses for keratoconus

**DOI:** 10.1371/journal.pone.0263506

**Published:** 2022-02-08

**Authors:** Takashi Kumanomido, Kazutaka Kamiya, Masahide Takahashi, Tatsuhiko Tsujisawa, Hideki Hayakawa, Wakako Ando, Yoshikazu Utsumi, Nobuyuki Shoji

**Affiliations:** 1 Department of Ophthalmology, Kitasato University Hospital, Kanagawa, Japan; 2 Department of Visual Physiology, School of Allied Health Sciences, Kitasato University, Kanagawa, Japan; University of Missouri-Columbia, UNITED STATES

## Abstract

Aspherical- and multi-curve rigid gas-permeable hard contact lenses (HCLs) have a flattened curve in the peripheral zone and are mostly used for patients with keratoconus who cannot wear glasses, soft contact lenses, or spherical HCLs. In this retrospective study, a total of 95 eyes of 77 patients who used aspherical- or multi-curve HCLs (mean age: 40.0 ± 11.0 years) were evaluated. This study examined the types of aspherical- and multi-curve HCLs, best-corrected visual acuity (BCVA) values before and after wearing HCLs, the association with the Amsler-Krumeich classification, duration of wear, corneal/conjunctival disorder, and the frequency of changing HCLs. There were 78 eyes that used aspherical-curve HCLs and 17 that used multi-curve HCLs. BCVA significantly improved from 0.42 logMAR to 0.06 logMAR after wearing either form of HCL. The Amsler-Krumeich classification showed that aspherical-curve HCLs were commonly used for patients with stage 2 keratoconus, and multi-curve HCLs were commonly used for stage 4 patients. The BCVA values were worse when the disease stage was more severe (stages 3 and 4) regardless of HCL type. The mean base curve of the lenses was steeper in multi-curve HCLs than in aspherical-curve HCLs. The more severe the disease stage, the steeper the base curve in both aspherical- and multi-curve HCLs. The duration of wear significantly improved from 2.1 h to 10.2 h, and corneal/conjunctival disorder similarly improved. The mean frequency of changing HCL types was 1.1 times. This study suggests that a flat peripheral curve design with aspherical- and multi-curve HCLs is useful for patients with keratoconus.

## Introduction

Keratoconus is a bilateral corneal dysmorphic disorder in which the center of the corneal stroma is thinned and protrudes anteriorly. The changes in corneal shape are accompanied by abnormalities in visual function. The cause of the disease remains unknown, despite various reports [1–3]. The incidence has been reported to be 1 in 2,000 [4]. However, with the advancement of corneal shape analysis technology, cases that were considered to be mild astigmatism can currently be diagnosed as keratoconus, and the incidence has been more recently reported to be 1 in 375 [5].

The normal corneal structure has an elliptical shape that mildly steepens in the central corneal zone and is almost uniformly flat between the intermediate corneal zone and the peripheral corneal zone. The value of the radius of corneal curvature, the index of the corneal shape, changes evenly from the central corneal zone through the peripheral corneal zone. In eyes with keratoconus, the corneal apex can be in the lower region rather than in the central region, and the central cornel apex is severely protruded. Consequently, the corneal shape may change unevenly. In early-stage keratoconus, the corneal shape is close to normal, and the degree of astigmatism is small.

Visual correction for early-stage keratoconus is possible with glasses and soft contact lenses (SCLs); however, rigid gas-permeable hard contact lenses (HCLs) are usually indicated [6]. Among several HCLs, spherical HCLs have a uniform structure with a central optical zone radius of curvature (base curve; BC) through the peripheral and bevel zones. During early-stage keratoconus, astigmatism can be corrected using spherical HCLs. However, in advanced-stage keratoconus, the degree of astigmatism often increases due to uneven corneal shape and renders the use of spherical HCLs more difficult.

Aspherical- and multi-curve HCLs are designed with a radius of curvature in the peripheral zone that is non-uniformly prepared relative to the BC. These HCLs can correct the vision even in advanced keratoconus [7–9]. HCLs without a flat peripheral zone will usually have a poor HCL fit. However, some reports have not discussed the curve design of the peripheral or bevel zone [10, 11], which needs to be addressed to accurately determine the effect of the curve design on keratoconus treatment with HCLs.

This study aimed to retrospectively assess the outcomes of treatment with aspherical- or spherical multi-curve HCLs (hereafter, multi-curve HCLs) with flat curves in the peripheral zone for patients with keratoconus for whom vision could not be corrected with glasses, SCLs, or spherical HCLs.

## Materials and methods

### Study design

The study was approved by the Institutional Review Board of Kitasato University Hospital (B19-365) and was performed in accordance with the Declaration of Helsinki. The Institutional Review Board waived the requirement for informed consent for the review of the clinical charts.

The study comprised a total of 95 eyes of 77 consecutive patients with keratoconus (65 eyes of 54 males and 30 eyes of 23 females; mean age, 40.0 ± 11.0 years) who were diagnosed at Kitasato University Hospital between January 2016 and December 2018. The overall follow-up period was 19.7 ± 7.9 months, with mean follow-up periods for aspherical- and multi-curve HCLs of 17.5 ± 8.8 and 21.0 ± 7.2 months, respectively.

The indication criteria were patients who could not wear glasses, SCLs, or spherical HCLs. In contrast, exclusion criteria were inflammatory eye disease, glaucoma, cataract, retinal disease, or giant papillary conjunctivitis.

For the diagnosis of keratoconus, the corneal shape was measured using corneal anterior segment optical coherence tomography (AS-OCT) (TOMEY, Nagoya, Japan). The data are presented using an absolute scale. Clinical findings, such as protrusion and thinning of the corneal apex, Vogt’s striae, and Fleischer’s ring, were observed using a slit-lamp microscope (Haag-Streit International, Bern, Switzerland).

For data measurement, the best-corrected visual acuity (BCVA) in the logarithm of the minimum angle of resolution (logMAR) was measured before and after wearing aspherical- or multi-curve HCLs. The outcomes of wearing aspherical- and multi-curve HCLs were analyzed. Subsequently, we analyzed the association of the severity of keratoconus with the BCVA values and the outcomes of wearing HCLs. Keratoconus severity was classified using the Amsler-Krumeich classification system [12]. The mean duration of lens wear, complications with corneal/conjunctival disorders, and the frequency of changing HCL types were used as clinical findings.

### Lens design and fitting

**Fig 1:**
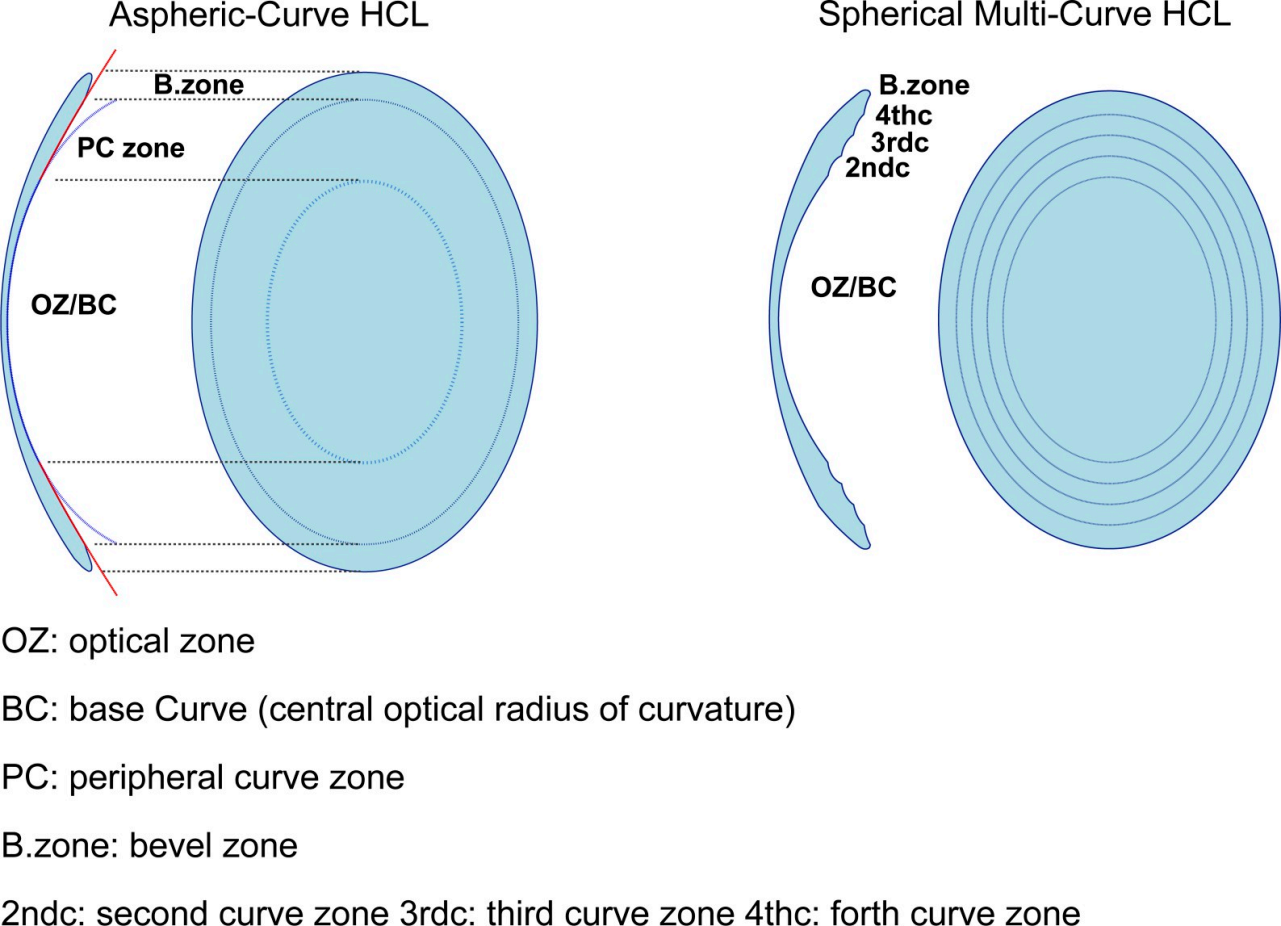
Rigid gas-permeable hard contact lens (HCL) design.

Aspherical-curve HCLs (HI SANSOα-Aspheric lens^®^) and multi-curve HCLs (HI SANSOα-Multi-curve lens^®^) used in this study were obtained from Rainbow Optical Contact Lens Corporation (Tokyo, Japan). Aspherical-curve HCLs have an optical zone with a spherical BC and a peripheral zone with an aspheric structure designed using a conchoid curve. The constant that determines the shape of a curve is defined as eccentricity (E); the larger the E value, the flatter the peripheral zone curve. We designed three types of aspherical-curve HCLs characterized by an E value of 3, 4, or 5 of the peripheral zone curve (Fig 1, Table 1).

**Table 1:** Specifications of rigid gas-permeable hard contact lenses.

Aspherical curve HCL												
			E3 type				E4 type			E5 type		
**Optical diameter, mm**			5.0				5.0			6.0		
**BC, mm**			6.00–8.70				5.50–8.70			6.0–8.70		
**Lens size, mm**			9.6				9.6			9.8		
**Dk value**			60 ×10^−11^(cm^2^/s)∙(mL O_2_/(mL × mmHg)									
**Lens power, D**			-20.00 to +20.00 (0.25 D step)									
**Multi-curve HCL**												
				OZD, mm	BC, mm	2^nd^ curve, mm		3^rd^ curve, mm	4^th^ curve, mm		Bevel, mm	Lens size, mm
**3 level curve**	**Steep type**			6.0	5.00–8.00	BC +0.5		BC +1.0			0.60	8.8
	**Normal type**			6.0	5.00–7.00	BC +0.5		BC +1.0			0.70	8.8
	**Flat type**			6.0	5.00–6.90	BC +1.0		BC +1.5			0.70	9.0
**4 level curve**	**Normal type**			5.5	5.00–8.00	BC +0.5		BC +1.0	BC +1.5		0.45	8.8
	**Steep type**			5.5	5.00–7.00	BC +1.0		BC +1.5	BC +1.5		0.70	8.8
**Dk value**		60 ×10^−11^(cm^2^/s)∙(mL O_2_/(mL × mmHg)										
**Lens power, D**		0 to—20.00 (0.25 D step)										

E, eccentricity; OZD, optical zone diameter; BC, base curve; Dk, oxygen permeability: (diffusion coefficient) × (solubility); D, diopter.

Multi-curve HCLs are designed with several different spherical curves from the BC to the peripheral zone. They have a 3- or 4-level structure, including the BC, depending on the number of spherical curves in the peripheral zone. In this study, there were three types of 3-level multi-curve HCLs and two types of 4-level multi-curve HCLs, due to differences in the curve design of the peripheral zone. Multi-curve HCLs were developed by Dr. Yoshikazu Utsumi (Utsumi Eye Clinic, Yokohama, Japan) (Fig 1, Table 1).

HCL parameter selection, the radius of corneal curvature as measured by AS-OCT, and BCs of HCLs were compared to select trial lenses. After the trial lenses were worn, fluorescein patterns of HCL staining, fitting, centering, and movement using slit-lamp microscopy were assessed. HCL fitting on the cornea was performed based on a three-point-touch technique to determine the best HCL.

### Statistical analyses

Statistical analyses were performed using an add-in software for Microsoft Excel (Statcel 4; Microsoft, Redmond, WA). The normality of all data samples was first checked using the chi-squared distribution test. Since none of the data were normally distributed, the data were compared using the Wilcoxon signed-rank test and Mann–Whitney U test. The stages 1 to 4 keratoconus data were compared using one-way analysis of variance (ANOVA) when possible. The data are presented as means ± standard deviation, and a value of p <0.05 was considered statistically significant.

## Results

Of the 95 eyes, 78 (55 males, 23 females, age, 40.1 ± 13.8 years) wore aspherical-curve HCLs, and 17 (10 males, 7 females; age, 38.1 ± 11.2 years) wore multi-curve HCLs (Table 2).

**Table 2:** Patient demographics of the study population.

	All	Aspherical-curve HCL	Spherical multi-curve HCL	p*-*value
**Number of eyes**	95	78	17	
**Male:female**	65:30	55:23	10:7	0.238
**Age (years)**	40.0 ± 11.0	40.1 ± 13.8	38.1 ± 11.2	0.632
**range (years)**	17–71	17–71	20–58	
**Follow-up period (months)**	19.7 ± 7.9	17.5 ± 8.8	21.0 ± 7.1	0.654
**BCVA (logMAR)**	0.06 ± 0.16	0.06 ± 0.13	0.05 ± 0.06	0.943
**Base curve (mm)**	6.59 ± 0.6	7.20 ± 0.5	6.13 ± 0.4	<0.001
**Wear time (hours)**	10.1 ± 3.7	10.2 ± 3.7	11.3 ± 3.1	0.562
**(Pre-used Aspherical, multi-curve)**	(2.1 ± 4.2)	(2.1 ± 4.0)	(2.2 ± 5.2)	0.016
**Mean keratometry (D)**	58.1 ± 4.4	57.3 ± 4.8	62.5 ± 5.5	<0.001
**Corneal astigmatism (D)**	4.6 ± 3.2	4.6 ± 3.5	4.7 ± 1.2	0.284
**Central corneal thickness (μm)**	473.3 ± 68.7	479.5 ± 68.9	440.2 ± 60.1	0.454
**Minimum corneal thickness (μm)**	433.0 ± 82.2	445.6 ± 79.1	360.2 ± 70.5	0.002

HCL, hard contact lens; BCVA, best-corrected visual acuity; logMAR, logarithm of the minimum angle of resolution; D, diopter.

The BCVA significantly improved from 0.42 ± 0.45 to 0.06 ± 0.16 logMAR in the whole study population (p < 0.001; Wilcoxon signed-rank test). The BCVA in the aspherical-curve HCL group improved from 0.37 ± 0.42 to 0.06 ± 0.13 logMAR, and those in the multi-curve HCL group improved from 0.66 ± 0.55 to 0.05 ± 0.06 logMAR. There was no significant difference in the BCVA between aspherical- and multi-curve HCL groups (p = 0.943; Mann–Whitney U test).

The overall BC was 6.59 ± 0.6 mm in the whole study population. The BC in the aspherical-curve HCL group was 7.20 ± 0.5 mm, and that in the multi-curve HCL group was 6.13 ± 0.4 mm (Table 2).

According to the Amsler-Krumeich classification, the BCVA was worse in more severe stages (stages 3 and 4) than in mild stages (stages 1 and 2) in both aspherical- and multi-curve HCL groups, with a significant difference observed (p = 0.017; ANOVA) (Table 3).

**Table 3:** Best-corrected visual acuity for aspherical- and spherical multi-curve hard contact lenses according to the Amsler-Krumeich classification.

	Stage				p*-*value
	1	2	3	4	
**Aspherical-curve HCL**	0.006 ± 0.13	0.02 ± 0.05	0.09 ± 0.11	0.14 ± 0.20	0.017 (ANOVA)
	1	2	3	4	
**Spherical multi-curve HCL**	No wear	0.02 ± 0.06	0	0.13 ± 0.12	<0.001 (Stage 2 vs. 4, Mann-Whitney U test)

ANOVA, analysis of variance; HCL, hard contact lens.

The E4 type lenses were mostly used in the aspherical-curve HCL group, and the 4-level steep-type lenses were mostly used in the multi-curve HCL group (Table 4).

**Table 4:** Types of aspherical- and spherical multi-curve hard contact lenses used for keratoconus.

	Lens type	Cases / eyes
**Aspherical-curve HCL 78 eyes (82%)**	E3	3 / 4
	E4	50 / 65
	E5	8 / 9
**Spherical multi-curve HCL 17 eyes (18%)**	3-level normal	4 / 4
	3-level steep	1 / 2
	3-level flat	3 / 3
	4-level normal	2 / 2
	4-level steep	6 / 6
**Total**		77 / 95

HCL, hard contact lens; E, eccentricity.

Aspherical-curve HCLs were used in 13 (17%) eyes with stage 1 keratoconus, 30 (38%) with stage 2, 12 (15%) eyes with stage 3, and 18 (23%) eyes with stage 4. Multi-curve HCLs were used in 3 (18%) eyes with stage 2 keratoconus, 1 (6%) eye with stage 3, 10 (59%) eyes with stage 4, and none for stage 1 (0%).

The more severe the stage, the steeper the BC was in eyes that used both aspherical- and multi-curve HCLs (p = 0.0002; ANOVA). The mean BC in the multi-curve HCL group was steeper than that in the aspherical-curve HCL group, especially in stages 2 and 4 (Table 5).

**Table 5:** Lens base curves of aspherical- and spherical multi-curve HCLs according to the Amsler-Krumeich classification.

		Stage				p-value
	**Aspherical-curve HCL**	1	2	3	4	
**BC (mm)**		7.4 ± 0.4	7.2 ± 0.3	7.1 ± 0.4	6.9 ± 0.5	<0.001 (ANOVA)
	**Spherical multi-curve HCL**	1	2	3	4	
		No wear	6.2 ± 0.3	6.9	6.0 ± 0.3	<0.001 (Stage 2 vs. 4, Mann-Whitney U test)

ANOVA, analysis of variance; BC, base curve; HCL, hard contact lens.

The duration of HCL wear significantly increased from 2.1 ± 4.2 h to 10.1 ± 3.7 h in the entire study population (p < 0.001; Wilcoxon signed-rank test) (Table 2). The total incidence of corneal and conjunctival epithelial disorders decreased from 37.9% (n = 36) to 16.8% (n = 16), and the total incidence of allergic conjunctivitis decreased from 22.1% (n = 21) to 4.2% (n = 4). The HCLs were changed in 26 of 95 eyes at a mean frequency of 1.1. The changes in the lens power and the lens curve were required in 9 eyes due to poor visual correction and 20 eyes due to uncomfortable HCL fit (12 eyes for a flat fit and 8 eyes for a steep fit).

At the final follow-up, we found significant differences in mean keratometry (p = 0.002; Mann-Whitney U test) and minimum corneal thickness (p = 0.005) between the two groups; however, no significant differences in corneal astigmatism or central corneal thickness were observed (Table 6).

**Table 6:** Corneal shape parameters in the aspherical- and spherical multi-curve HCL groups at the final follow-up.

	All	Aspherical-curve HCL	Spherical multi-curve HCL	p*-*value
**Mean keratometry (D)**	52.5 ± 7.7	51.1 ± 6.8	62.4 ± 5.9	0.002
**Corneal astigmatism (D)**	4.9 ± 3.9	4.7 ± 3.3	6.7 ± 7.0	0.769
**Central corneal thickness (μm)**	474.3 ± 63.3	479.8 ± 63.0	434.9 ± 54.4	0.309
**Minimum corneal thickness (μm)**	435.8 ± 78.7	447.0 ± 69.3	348.1 ± 92.6	0.005

HCL, hard contact lens; D, diopter.

## Discussion

In this study, we designed aspherical- and multi-curve HCLs with flat peripheral zones to treat keratoconus. Our findings indicate that a flat peripheral curve design with aspherical- and multi-curve HCLs is clinically beneficial, especially for patients with advanced keratoconus, resulting in a decrease in the number of surgical cases for penetrating keratoplasty or deep anterior lamellar keratoplasty. We used the AS-OCT for the diagnosis and the grade classification of keratoconus. Maeda et al. [13] and Naderan et al. [14] used corneal topography and corneal tomography to assess the corneal shape, respectively. Naderan et al. [14] reported that the results of the corneal shape analysis with Pentacam (OCULUS, Arlington, WA) were highly associated with the Amsler-Krumeich classification. Kamiya et al. [15] reported that the results of AS-OCT corneal tomography were highly associated with the classification of the disease as well.

The current findings demonstrated a significant BCVA improvement during wearing aspherical- and multi-curve HCLs. The mean durations of HCL wear were 10.2 ± 3.7 h for aspherical-curve HCLs and 11.3 ± 3.1 h for multi-curve HCLs (Table 2); however, the BCVA was worse in more severe stages (Table 3). The causes were considered to be poor corneal shape and corneal opacity.

Aspherical-curve HCLs were often used for patients in keratoconus stages 2 and 3, whereas multi-curve HCLs were often used for those in stage 4. We assume that this is because as keratoconus progresses, the corneal cone protrusion worsens. When aspherical-curve HCL is fitted to a protruded corneal cone, there can be a space between the HCL and the cornea, resulting in a poor HCL fit. On the contrary, even when the corneal cone protrusion worsens, there is less space between the multi-curve HCL and the cornea, resulting in a good HCL fit since the peripheral zone has a multi-level curve. Therefore, it is reasonable that multi-curve HCLs are more suitable for advanced keratoconus.

The BC of both aspherical- and multi-curve HCLs steepens in severe stages due to the anterior protrusion of the cornea. The steeper BC of the multi-curve HCLs compared to aspherical-curve HCLs, even in the same stage, may be associated with the position of the corneal cone. We suggest that the BC in the central zone be flat when a corneal cone is present in the lower region, whereas the BC should be steep when the cone is in the central zone (Table 5).

Previous studies on aspherical-curve HCLs have been reported by Yanai et al. [16] and Kazanci et al. [17]. In the report by Yanai et al., the E value of the peripheral-zone curve design was small, and HCLs were not used for severe cases [16]. In this study, aspherical-curve HCLs were used for stages 3 and 4. Kazanci et al. reported poorer BCVA outcomes than those in our study [17]. Our aspherical-curve HCLs may have achieved better BCVA than those in previous studies because of the larger eccentricity (E) values in the peripheral-zone curve design, resulting in a good HCL fit (Table 7).

**Table 7:** Previous studies on aspherical-curve rigid gas-permeable hard contact lens for keratoconus.

	Current.	Yanai et al. (2010)	Kazanci et al. (2014)
**Lens design (Product name)**	Aspherical tri-curve (HI SANSOα Aspherical lens^®^)	Aspherical tri-curve (Aphex KC^®^)	Aspherical (Boston Eqalens^®^) (Boston7^®^)(CFKE^®^)
**Oxygen permeability (Dk value)**	60 × 10^−11^	61.3 × 10^−9^ (Dk/t)	Not listed
**Optical zone diameter (mm)**	5.0–6.0	5–7	Not listed
**Lens size (mm)**	9.6–9.8	8.4–9.6	Not listed
**Curve design (E)**	Optical zone: E 0	Optical zone: E 0.4–0.45	Not listed
	Peripheral zone: E 3,4,5	Peripheral zone: E 0.6	
**Number of eyes**	78	29	155
**Follow-up (months)**	17.5 ± 8.8	40.9 ± 19.7	12
**Best-corrected visual acuity (logMAR)**	0.06 ± 0.13	0.2 better	0.75–0.8 (median) (decimal)
**Wear time (hours)**	10.2 ± 3.7	12.6 ± 3.2	Boston Eqalens^®^
			8.0 ± 1.39
			Boston7^®^
			7.9 ± 1.19
			CFKE^®^
			8.7 ± 2.2
**Success rate**	98.7%	86.2%	Not listed
**Grade of keratoconus**	Stage 1, 13 eyes	<45 D, 5 eyes	<45 D, 0 eye
	Stage 2, 30 eyes	45≤, <52 D, 10 eyes	45≤, <52 D, 92 eyes
	Stage 3, 12 eyes	52≤, <62 D, 7 eyes	52≤, <62 D, 57 eyes
	Stage 4, 18 eyes	≥62 D, 7 eyes	>62 D, 4 eyes
	Not classified, 5 eyes	(Mean K)	(Mean K)

Dk, (diffusion coefficient) × (solubility) (cm^2^/s) (mL O_2_/ (mL × mmHg); t, thickness (cm), E, eccentricity; D, diopter; logMAR, logarithm of the minimum angle of resolution; Mean K, mean keratometry.

In previous studies on multi-curve HCLs, the HCLs were not designed to have a flat peripheral zone curve; however, the BCVA, duration of HCL wear, and HCL wearing success rates were better than those obtained in this study [17–19]. The reason for the poor result of multi-curve HCLs in our study may be that most multi-curve HCLs were used for stage 4 cases. Nevertheless, the BCVA was 0.05 ± 0.06, and multi-curve HCLs were appropriately used (Table 8).

**Table 8:** Summary of previous studies on multi-curve rigid gas-permeable hard contact lens for keratoconus.

	Current	Yanai et al. (2013)	Kazanci et al. (2014)	Lee et al. (2004)
**Lens design (Product name)**	Multi-curve (HI SANSOα Multi-curve lens^®^)	Multi-curve (TwinbelⅡ^®^)	Multi-curve (Rose K^®^)	Multi-curve (YK Lens^®^)
**Oxygen permeability (Dk value)**	60 × 10^−11^	12.1 × 10^−11^	100 × 10^−11^	47 × 10^−11^
**Optical zone diameter (mm)**	5.5–6.0	7.0–8.0	Not listed	Not listed
**Lens size (mm)**	8.8–9.0	8.5–10.0	Not listed	8.7
**Peripheral Curve design (mm)**	2^nd^ BC + 0.5	BC +0.3–0.7	Not listed	2^nd^ BC + 0.2
	3^rd^ BC + 1.0, 1.5			3^rd^ BC + 0.4
	4^th^ BC + 1.5			4^th^ BC + 0.6
**Number of eyes**	17	9	74	72
**Mean follow-up (months)**	21.0 ± 7.21	13.3 ± 1.4	12	11.4
**Best-corrected visual acuity (logMAR)**	0.05 ± 0.06	0.01 ± 0.40	0.8 (median) (decimal)	20/30
**Wear time (hours)**	11.3 ± 3.1	12.6 ± 3.2	9.7 ± 0.88	11.9
**Success rate**	82.3%	100%	Not listed	95%
**Grade of keratoconus**	Stage 1, 0 eye	<45 D,	<45 D,	47.6 ± 4.5 D
	Stage 2, 3 eyes	4 eyes	0 eye	(Max K)
	Stage 3, 1 eye	45≤, <52 D,	45≤, <52 D,	
	Stage 4, 10 eyes	1 eye	42 eyes	
	Not classified, 3 eyes	52≤, <62 D,	52≤, <62 D,	
		3 eyes	31 eyes	
		>62 D,	>62 D,	
		1 eye	1 eye	
		(Mean K)	(Mean K)	

Dk, (diffusion coefficient) × (solubility): (cm^2^/s) (mL O2/(mL × mmHg); logMAR, logarithm of the minimum angle of resolution; D, diopter; Mean K, mean keratometry; Max K, maximum keratometry.

Higher-order aberrations and contrast sensitivity function were evaluated during HCL wear in some previous studies. Yanai et al. [18] reported no significant difference in higher-order aberrations before and during HCL use. In contrast, Negishi et al. [20] reported that ocular higher-order aberrations increased during HCL wear for keratoconus even when corrected visual acuity was good. Similarly, Wei et al. [21] reported that contrast sensitivity significantly decreased. Nevertheless, it is still necessary to compare higher-order aberrations and contrast sensitivity function between the aspherical- and multi-curve HCL groups according to the Amsler-Krumeich classification.

Regarding vision correction for keratoconus, not only HCLs, but also asymmetric SCLs [22], scleral lenses [23], and hybrid contact lenses with HCL and SCL structures in the optical and peripheral zones, respectively, have been developed [24]; however, these lenses have not been widely used in Japan.

Kamiya et al. [25] reported good outcomes of toric posterior chamber phakic intraocular lens implantation for mild keratoconus during the 3-year observation period [26]. However, the indication for this surgery is still limited for mild and non-progressive keratoconus, which is largely different from that in our study population, including advanced keratoconus.

There are at least two limitations to this study. Firstly, this study was performed at a single center, and, thus, the sample size was relatively small. Secondly, it is still difficult to objectively evaluate the optimal means of HCL wearing exclusively based on the clinical appearances. Elbendary et al. [27] measured the tear film thickness between the HCL and cornea using the AS-OCT. However, we are currently not available for the quantitative measurements of the fluorescein pattern or the HCL movement. In addition, aspherical- and multi-curve HCLs are indicated for patients with irregular corneal shapes, such as keratoconus; however, they are not indicated for other populations with normal corneal shapes.

## Conclusions

A flat peripheral curve design with either aspherical- or multi-curve HCLs was clinically beneficial for patients with keratoconus. Multi-curve HCLs tended to be applied, especially in advanced keratoconus. Aspherical- and multi-curve HCLs may help obtain good visual outcomes for keratoconus without surgical interventions. Nevertheless, a further prospective comparative study with a large cohort of patients with keratoconus is necessary to confirm our findings.
